# Pancreatic Mass: Include Tuberculosis in the Differential Diagnosis

**DOI:** 10.7759/cureus.15430

**Published:** 2021-06-03

**Authors:** Darpan Sohni

**Affiliations:** 1 General Medicine, Osmania Medical College, Hyderabad, IND

**Keywords:** pancreas, pancreatic mass, tuberculosis, pancreatitis, pancreatic tuberculosis, differential diagnosis

## Abstract

Although abdominal tuberculosis (TB) is quite prevalent in endemic regions, involvement of the pancreas is considerably rare. We describe a case of pancreatic TB presenting as a pancreatic mass in a patient with abdominal pain and jaundice. Due to the similar presentation, it can easily be misinterpreted as a pancreatic neoplasm. Endoscopic ultrasound-guided fine needle aspiration (EUS-FNA) can help confirm the diagnosis in such cases by providing histopathological evidence of *Mycobacterium tuberculosis* infection. The patient made a remarkable recovery post anti-tuberculous therapy (ATT) initiation. This exceptional response of pancreatic TB to conservative management makes it imperative that the condition be diagnosed promptly to avoid any futile surgical interventions and associated complications. This can only be achieved if clinicians are aware of the diagnostic possibility of pancreatic TB presenting as a mass in the pancreas.

## Introduction

Tuberculosis (TB) of the pancreas is a remarkably unusual clinical entity; all the more so in immunocompetent individuals. Its clinical features are usually vague and non-specific. The radiological features mimic pancreatic malignancy in many cases and pancreatitis in others, allowing pancreatic TB to elude the diagnosis [1]. Herein we describe a case of isolated pancreatic TB presenting as a discrete mass in the pancreas in an immunocompetent host.

## Case presentation

A 52-year-old male presented to the casualty with complaints of abdominal pain and discomfort for three days. The pain was progressive, non-radiating, and predominantly in the epigastric region. He also complained of nausea for two days and one episode of vomiting. The patient denied any history of fever or diarrhea. The review of systems was positive for decreased appetite and weight loss of six months duration. The patient had a history of admission for acute pancreatitis four years prior for which he was treated conservatively. He had a 25-pack-year smoking history and consumed 12-24 ounces (350-700 ml) of beer daily for 15 years but quit alcohol after his previous admission. The patient had no comorbidities and was not on any medications. Family history was not significant.

The patient was hemodynamically stable. Clinical examination showed a thin and lean individual in mild distress. BMI was 20.2 kg/m2. Sclerae were icteric. Abdominal examination revealed moderate tenderness in the epigastric and umbilical regions. There was no guarding, rigidity, or rebound tenderness. The remainder of the physical examination was unremarkable.

Laboratory investigations were significant for a total bilirubin of 4.1 mg/dl, alanine aminotransferase of 132 IU/L, aspartate aminotransferase of 114 IU/L, and alkaline phosphatase of 362 IU/L. Mild elevations in serum lipase and amylase were noted - 186 U/L and 194 U/L, respectively. Autoimmune and viral workup was negative.

**Figure 1:**
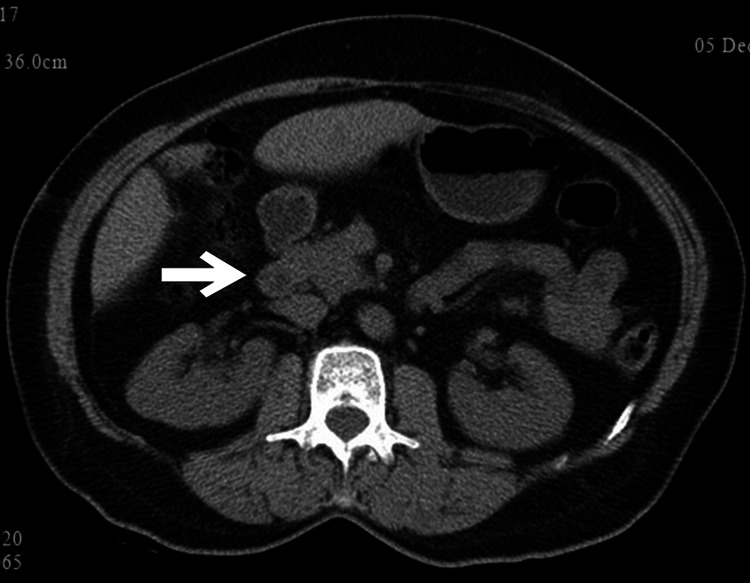
Plain CT image showing a homogeneous lesion in the pancreatic head.

Chest X-ray showed no abnormalities. CT of the abdomen showed a homogeneous mass at the pancreatic head with infiltration of the portal vein and enlarged peripancreatic lymph nodes. There was a mild narrowing of the proximal common bile duct with mild bilobar intrahepatic biliary dilatation (Figure 1).

**Figure 2:**
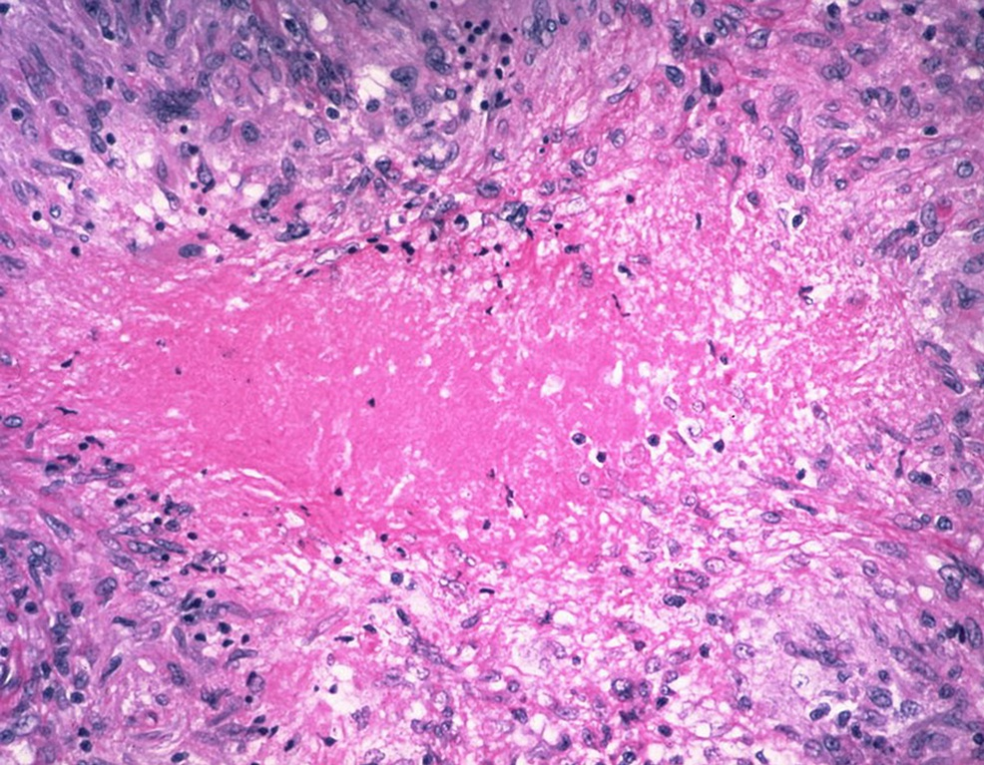
Hematoxylin and eosin-stained histological section showing granuloma with central necrosis.

Fine needle aspiration (FNA) of the pancreatic mass and periportal lymph node was performed under ultrasound guidance. Histopathological examination (HPE) of the specimens revealed the focus of epithelioid cells with coagulation necrosis consistent with granulomatous inflammation (Figure 2).

The diagnosis of TB was confirmed when the tissue samples showed acid-fast bacilli (AFB) and the cultures grew *Mycobacterium tuberculosis*. Sputum samples sent for AFB staining and Gene-Xpert test came back negative. The patient was started on anti-tuberculous therapy (ATT) with first-line agents. The patient regained his appetite and weight and was doing well six months post-treatment. A follow-up CT taken almost seven months after treatment initiation showed resolution of the pancreatic lesion.

## Discussion

Although abdominal TB is quite prevalent in endemic regions, its occurrence in the pancreas is exceedingly uncommon. The majority of the pancreatic TB cases that have been described in the literature were seen in the setting of disseminated disease and/or in immunodeficient subjects. Ours was a rare case of isolated pancreatic TB in a robust individual. Furthermore, the detection of AFB in the biopsy sample was unanticipated since most of the abdominal TB cases are paucibacillary [2].

Pancreatic TB usually presents with nonspecific symptoms such as abdominal pain, jaundice, vomiting, anorexia, or weight loss [3]. CT and MRI frequently show a pancreatic mass and almost half the cases exhibit involvement of peripancreatic lymph nodes [4]. Since pancreatic neoplasms have analogous presentations, the ambiguity often leads to unwarranted surgical resection of the pancreatic mass. Endoscopic ultrasound-guided fine-needle aspiration (EUS-FNA) is the diagnostic modality of choice for pancreatic TB [5]. It is less invasive, carries lower risks when compared to laparotomy, and can provide a conclusive diagnosis by providing samples for HPE. The presence of caseating granulomatous inflammation is the commonest finding on HPE [2].

Medical management with ATT remains the cornerstone of the treatment of TB. Most patients respond favorably to conservative management and a complete recovery is possible with six to twelve months of treatment. Hence, it is imperative that the condition be diagnosed promptly and the patient started on ATT sooner rather than later.

Once unheard of, cases of pancreatic TB are being increasingly reported today. Physicians have to be aware of its existence and show a high index of suspicion when dealing with an obscure pancreatic mass; more notably so in endemic regions and immunodeficient individuals. This will help shorten the time from presentation to diagnosis and also spare patients from risky and unwarranted surgical procedures [4].

## Conclusions

Pancreatic TB is a rare disease. Nevertheless, clinicians should consider it a diagnostic possibility when evaluating a patient with a pancreatic mass. Doing so could ensure an early diagnosis and help prevent any futile surgical interventions and associated complications. The disease is potentially curable and patients should have a timelier initiation of ATT for better treatment outcomes.

## References

[1] Pramesh CS, Heroor AA, Gupta SG, Krishnamurthy S, Shukla PJ, Jagannath P, DeSouza LJ (2003). Pancreatic tuberculosis: an elusive diagnosis. HPB.

[2] Veerabadran P, Sasnur P, Subramanian S, Marappagounder S (2007). Pancreatic tuberculosis-abdominal tuberculosis presenting as pancreatic abscesses and colonic perforation. World J Gastroenterol.

[3] Bhurwal A, Haq MM, Sapru S, Tortora M, Ramasamy D (2018). Isolated pancreatic tuberculosis mimicking pancreatic cancer: a diagnostic challenge. Case Rep Gastrointest Med.

[4] Panic N, Maetzel H, Bulajic M, Radovanovic M, Löhr J-M (2020). Pancreatic tuberculosis: a systematic review of symptoms, diagnosis and treatment. United European Gastroenterol J.

[5] Jemni I, Akkari I, Mrabet S, Jazia EB (2020). Isolated pancreatic tuberculosis mimicking pancreatic cancer in an immunocompetent host: an elusive diagnosis. Radiol Case Rep.

